# Perinucleolar Compartment (PNC) Prevalence as an Independent Prognostic Factor in Pediatric Ewing Sarcoma: A Multi-Institutional Study

**DOI:** 10.3390/cancers15082230

**Published:** 2023-04-10

**Authors:** Elizabeth Gonzalez, Atif A. Ahmed, Laura McCarthy, Katherine Chastain, Sahibu Habeebu, Marta Zapata-Tarres, Rocio Cardenas-Cardos, Liliana Velasco-Hidalgo, Celso Corcuera-Delgado, Rodolfo Rodriguez-Jurado, Lilia García-Rodríguez, Alejandro Parrales, Tomoo Iwakuma, Midhat S. Farooqi, Brian Lee, Scott J. Weir, Terrie G. Flatt

**Affiliations:** 1 Department of Pediatrics, Division of Hematology & Oncology, Children’s Mercy Hospital, University of Missouri-Kansas City School of Medicine, Kansas City, MO 64108, USA; eliglezdgz@gmail.com (E.G.); laura.mccarthy@unitypoint.org (L.M.); kate.m.chastain@gmail.com (K.C.); aparralesbriones@cmh.edu (A.P.); tiwakuma@cmh.edu (T.I.); 2 MD/PhD (PECEM) Program, Facultad de Medicina, Universidad Nacional Autónoma de México, Mexico City 04360, Mexico; 3 Department of Pathology, Seattle Children’s Hospital, Seattle, WA 98105, USA; atif.ahmed@seattlechildrens.org; 4 Department of Pathology & Laboratory Medicine, Children’s Mercy Hospital, University of Missouri-Kansas City School of Medicine, Kansas City, MO 64108, USA; smhabeebu@cmh.edu (S.H.); msfarooqi@cmh.edu (M.S.F.); 5 Research Coordination Mexican Institute of Social Security Foundation, Mexico City 06600, Mexico; mzapatatarres@gmail.com; 6 Departamento de Oncología Pediátrica, Instituto Nacional de Pediatría, Mexico City 04530, Mexico; oncoped_inp@hotmail.com (R.C.-C.); lilianavh@hotmail.com (L.V.-H.); 7 Departamento de Patología Pediátrica, Instituto Nacional de Pediatría, Mexico City 04530, Mexico; ctcorcuera@hotmail.com (C.C.-D.); rrrj60@hotmail.com (R.R.-J.); 8 Facultad de Medicina, Universidad de Monterrey, San Pedro Garza García 66238, Mexico; legr.29@gmail.com; 9 Department of Health Services and Outcomes Research, Children’s Mercy Hospital, University of Missouri-Kansas City School of Medicine, Kansas City, MO 64108, USA; blee@cmh.edu; 10 Department of Cancer Biology, University of Kansas Medical Center, Kansas City, KS 66103, USA; sweir@kumc.edu; 11 Department of Pharmacology, Toxicology and Therapeutics, University of Kansas Medical Center, Kansas City, KS 66103, USA; 12 Institute for Advancing Medical Innovation, University of Kansas Medical Center, Kansas City, KS 66103, USA

**Keywords:** perinucleolar compartment, Ewing sarcoma, microRNA, metastasis, biomarker

## Abstract

**Simple Summary:**

The perinucleolar compartment (PNC) plays an important role in tumorigenesis. Its presence has been correlated with poor prognosis and cancer metastasis. Ewing sarcoma (EWS) is the second most common bone cancer in children and young adults. Currently, there are no clinically relevant markers predictive of which patients with Ewing sarcoma will experience early relapse, and there are no effective targeted agents for this disease to date. Using samples from primary and metastatic tumors, immunohistochemical study of PNC demonstrates a higher PNC prevalence in metastatic sites compared to primary tumors, and that a high prevalence of PNC was correlated with a distinct microRNA profile and shorter disease-free survival, highlighting its utility as a clinically relevant predictive biomarker associated with tumor metastasis. PNC prevalence was higher in Hispanic patients, potentially demonstrating a biologic basis for the ethnic disparity in this tumor.

**Abstract:**

The perinucleolar compartment (PNC) is a small nuclear body that plays important role in tumorigenesis. PNC prevalence correlates with poor prognosis and cancer metastasis. Its expression in pediatric Ewing sarcoma (EWS) has not previously been documented. In this study, we analyzed 40 EWS tumor cases from Caucasian and Hispanic patients for PNC prevalence by immunohistochemical detection of polypyrimidine tract binding protein and correlated the prevalence with dysregulated microRNA profiles. EWS cases showed staining ranging from 0 to 100%, which were categorized as diffuse (≥77%, n = 9, high PNC) or not diffuse (<77%, n = 31) for low PNC. High PNC prevalence was significantly higher in Hispanic patients from the US (n = 6, *p* = 0.017) and in patients who relapsed with metastatic disease (n = 4; *p* = 0.011). High PNC was associated with significantly shorter disease-free survival and early recurrence compared to those with low PNC. Using NanoString digital profiling, high PNC tumors revealed upregulation of eight and downregulation of 18 microRNAs. Of these, miR-320d and miR-29c-3p had the most significant differential expression in tumors with high PNC. In conclusion, this is the first study that demonstrates the presence of PNC in EWS, reflecting its utility as a predictive biomarker associated with tumor metastasis, specific microRNA profile, Hispanic ethnic origin, and poor prognosis.

## 1. Introduction

Ewing sarcoma is the second most common bone cancer in children and young adults [1]. The disease is characterized by the presence of fusion genes resulting from chromosomal translocation in the FET protein family (e.g., EWSR1, FUS) gene and an ETS-transcription family gene (e.g., FLI1, ERG, ETV1/4, and FEV) [2]. Most patients (85%) have the EWS-FLI1 fusion gene, resulting from a t(11;22) chromosomal translocation [3,4]. Although surgical resection combined with chemotherapy and radiation improved the 5-year overall survival of localized Ewing sarcoma to 65–70%, the overall survival of metastatic Ewing sarcoma remains stagnant at <30%, and those that relapse early, defined as less than 2 years from initial diagnosis, continue to have a dismal prognosis [5,6,7]. Greater than 70% of patients will relapse within 2 years from the time of initial diagnosis, and the 5-year survival for those with early relapse is approximately 7% compared to those who relapse after 2 years after the time of initial diagnosis [8]. Currently, there are no clinically relevant predictive biomarkers for disease progression or early relapse, and there are no effective targeted agents for this disease to date.

Found peripherally in the nucleolus, the Perinucleolar Compartment (PNC) is a small nuclear body measuring between 0.25 and 4 µm in length, enriched with RNA and RNA-binding proteins, and plays an important functional role in RNA metabolism, including alternative splicing [9,10,11]. The structural integrity of the PNC is dependent upon its RNA and protein components and is mediated by the RNA-binding ribonucleoprotein, polypyrimidine tract binding (PTB) protein [10]. Studies have shown that PNC is highly prevalent in cancers, particularly metastatic tumors, whether in transformed cell lines or the patient’s tumor tissues. PNC prevalence in cancer, defined as the percentage of non-mitotic and non-apoptotic cells containing at least one PNC, is variable and ranges from 5 to 100% [11,12,13]. Importantly, the PNC is almost exclusively found in non-hematopoietic solid cancer cells [9,13]. One of the essential PNC components, the PTB protein, is regulated by its interaction with other proteins and RNA molecules including microRNAs such as microRNA-124 [14,15,16]. Detection of PTB by immunohistochemistry in tumor tissues serves as a biomarker for the presence of this structure [17]. Detection of microRNA associated with PTB may also serve as another indicator of PNCs.

The PNC has been identified in adult cancers as a biomarker for prognosis and metastasis [9]. Because the PNC is not present in normal healthy cells and does not involve crucial growth or tumor suppressor pathways, it becomes an ideal target for novel anti-cancer drugs. A novel agent such as ML246 (or metarrestin) can inhibit PNC assembly and thus could disrupt cellular processes that are crucial in maintaining a malignant phenotype [12,18,19]. While the PNC has been studied in various adult tumors, very little is known about its presence or function in pediatric tumors. The primary objective of our study is to demonstrate the presence of the PNC in pediatric EWS and correlate PNC prevalence with clinical outcomes, as well as the microRNA (miRNA) profile in these tumors.

## 2. Materials and Methods

### 2.1. Patients and Samples

We retrospectively reviewed the medical records of patients who were diagnosed and treated for pediatric EWS at Children’s Mercy Hospital (CMH) in Kansas City, MO and the Instituto Nacional de Pediatría (INP) in Mexico City, Mexico between May 2012 to May 2019. Study inclusion criteria included pathologic confirmation of EWS as a new diagnosis in patients < 18 years. The diagnosis of EWS was confirmed with histological and immunohistochemical studies in all patients. All the cases revealed small round cell morphology with strong diffuse membranous CD99 immunoreactivity and negative immunoreactivity for other biomarkers relevant to the differential diagnosis. Cases with atypical spindle cell morphology, myxoid stroma, or aberrant CD99 immunoreactivity have been excluded. Cytogenetic and molecular tests for *EWSR1* gene rearrangement were performed on all patients from Children’s Mercy Hospital and were confirmatory. However, molecular tests were not reviewed in cases from Mexico. Patients with prior malignant disorder, chemotherapy, or radiation therapy, and those whose archived tissue was not available were excluded. We have retrieved and utilized archived formalin-fixed paraffin-embedded (FFPE) tumor blocks to determine PNC prevalence and miRNA profile. We have reviewed patients’ medical charts for clinical data that included demographics, overall survival, and disease-free survival. The study was approved by the respective Institutional Review Boards at CMH and INP. The IRB at both sites granted an exemption for consent/assent given the study is retrospective. Fourteen patients were from the INP and 26 from CMH. Children treated at the INP are of Mexican ancestral origin and their tumor samples (including H&E-stained slides and immunostains) were re-reviewed at CMH for diagnosis confirmation. All patients (both with localized and metastatic disease at time of diagnosis) at CMH and INP received therapy per Children’s Oncology Group (COG) AEWS0031 or EURO EWING 2012 protocols using interval compression therapy: induction [neo-adjunctive therapy consists of six two-week cycles of vincristine, doxorubicin, cyclophosphamide (VDC), alternating with ifosfamide and etoposide (I/E)], followed by definitive local control (surgery, radiation therapy, or a combination of the two modalities) and 8 additional cycles of VDC-IE. No patient received an autologous stem cell transplant. Patients were followed for an average of 31.9 months (range: 1 to 121 months; median: 20 months).

### 2.2. Immunohistochemistry

Automated immunohistochemistry on all samples was performed at CMH. FFPE sections were stained with a monoclonal antibody to PTB, clone SH54 (ThermoFisher Scientific, Waltham, MA, USA), using a Leica Bond autostainer (Leica Biosystems, Buffalo Grove, Illinois) and a polymer refine detection system. After heat antigen retrieval, the antibody was applied at a 1:100 dilution in a low pH buffer. Deparaffinized slides were incubated with a primary antibody, secondary antibody, and a polymer conjugate. A single brown color staining was visualized by incubation with DAB chromogen. To visualize the characteristic granular nuclear staining, the hematoxylin counterstain step was omitted. Breast carcinoma tissue served as a positive control. Stained sections were analyzed and graded for the extent of staining, which was reported as a percentage of stained cells in viable areas (from 0 to 100%). PNC staining prevalence was determined by 3 independent reviewers. Immunohistochemistry was performed on 48 paraffin-embedded tissue blocks, including 39 from the primary untreated tumor, 5 after neo-adjunctive treatment, and 4 from metastatic sites.

### 2.3. NanoString miRNA Profiling

Nucleic acid was retrieved from 23 FFPE EWS tumor tissue scrolls. After RNA extraction, a Nanostring nCounter was used to quantify the expression of 827 human miRNAs that have been sequenced with high confidence or have been found to be clinically relevant (nCounter Human v3 miRNA Expression Assay Kit) in tumor samples from de-identified patients. NanoString miRNA normalization was performed with background subtraction set to 30 and selecting the top 100 miRNA transcripts using the geometric mean algorithm to create a normalization factor. Samples with high variability (percent CV over 90%) were eliminated for downstream analysis. Normalized data were then imported into ROSALIND software ^®^ (https://rosalind.bio/ (accessed on 16 February 2022)), with a HyperScale architecture developed by ROSALIND, Inc. (San Diego, CA, USA). Read distribution percentages, violin plots, identity heatmaps, and sample MDS plots were generated as part of the QC step in ROSALIND. Fold changes and *p*-values for differential expression comparisons were generated using the *t*-test method. Clustering of miRNA for the final heatmap of differentially expressed miRNA was performed using the PAM (Partitioning Around Medoids) method using the fpc R library [20]. The top targeted gene predictions, validated genes, and related drugs and diseases were analyzed using the multiMiR R library [21]. miRNA secondary structures were calculated and visualized using the ViennaRNA software [22].

### 2.4. Statistical Analysis

We utilized the Wilcoxon Rank-Sum and Kruskall–Wallis tests to compare PNC proportions based on gender, age, race/ethnicity, tumor volume, size, necrosis, and localized versus metastatic disease at diagnosis. An ROC analysis was performed using PNC prevalence and our primary relapse and/or progression outcome variable, including calculating Youden’s J statistic to determine an optimal cut-point for attributing “low” vs. “high” PNC.

Kaplan–Meier curves were stratified by initial localized/metastatic status to better understand how the relationship between disease-free survival and PNC may be confounded by metastasis. Cox proportional hazard models were completed to evaluate the relationship between the patient relapse/progression outcome with PNC prevalence and metastatic status. Percentile-based bootstrapped confidence limits, using 5000 iterations, for these Cox models are reported. Statistical analyses were performed using R version 4.2.2 (R Core Team; Vienna, Austria).

To evaluate the relationship between PNC and miRNA profiles, we categorized PNC prevalence as low or high (based on a 77% cut-off) and compared the miRNA profiles of the two groups. Statistical tests of differences were calculated with fold change and student *t*-test.

## 3. Results

### 3.1. Data for Patient Race and Ethnicity as well as Tumor Metastasis and Recurrence

We examined 40 patients diagnosed with Ewing Sarcoma from May 2012 to May 2019 at Children’s Mercy Hospital in Kansas City, Missouri, and the Instituto Nacional de Pediatría (INP) in Mexico City, Mexico that met the inclusion criteria. Fourteen patients were from the INP and 26 from CMH. The mean age was 10.28 years (1–18 years), and 55% of patients were female. Race and ethnicity were as follows: Caucasians from CMH represented 48% (n = 19) while Hispanics were 15% (n = 6), and Asians 2.5% (n = 1). Mexicans from the INP represented 35% (n = 14) of the total population. Fifty-five percent (n = 16) of the patients presented with metastatic disease at initial diagnosis and of those, 31.2% (n = 5) had pulmonary metastatic disease, 37.5% (n = 6) had pulmonary and other distant metastatic sites, and 31.2% (n = 5) had extrapulmonary disease (bone and other organs). Seventy percent had either progression, relapse, or both (n = 28) and 53% died (n = 22) (Table 1). Patients survived for a mean period of 25 months (range of 7 to 43 months).

### 3.2. Correlation of High PNC Prevalence with Disease Outcome

**Figure 1:**
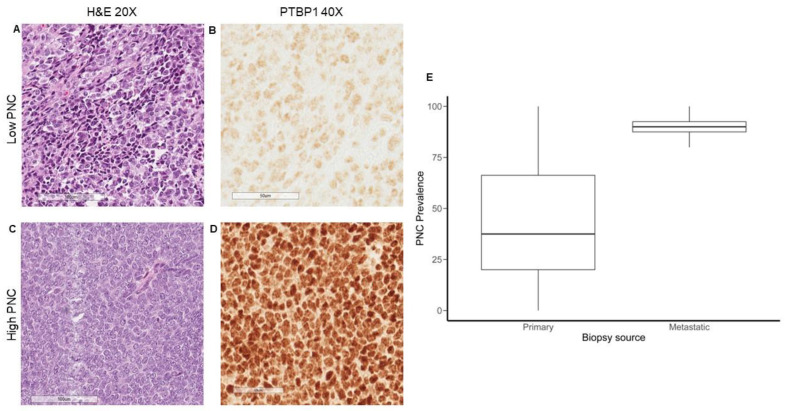
Prevalence of PNC in EWS tumor samples. Immunohistochemistry for PNC reveals distinct granular nuclear staining. While EWS tumors have the same histology (H&E X20 columns (**A**,**C**)), they exhibit variable PNC staining that can be categorized as low (**B**) to high (**D**). (**B**) revealed less intensity and more restricted staining distribution compared to (**D**). The biopsy source demonstrates a higher PNC prevalence at metastatic sites from patients with relapse (**E**).

Staining for the PNC was identified as granular nuclear brown staining (Figure 1B,D). Staining intensity was uniform in most of the tumors and the percentage of stained cells was calculated and reported. Immunostaining of greater than 10% of tumor cells was required for scoring as a positive case. Positive staining was identified in 35/40 or 87.5% of EWS samples and was further classified as follows: 0 or scant staining ≤ 10% (n = 5), 11 to 25% (n = 13), 26 to 50% (n = 8), >50–74% (n = 5), and ≥75% (n = 9). 

**Figure 2:**
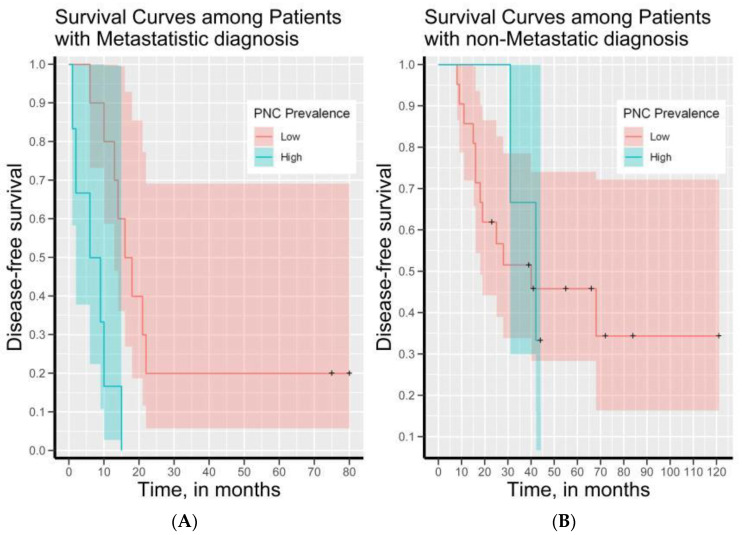
Kaplan–Meier analysis revealing poor progression-free survival among patients with metastatic diagnosis (**A**) and among patients with non-metastatic diagnosis (**B**).

**Figure 3:**
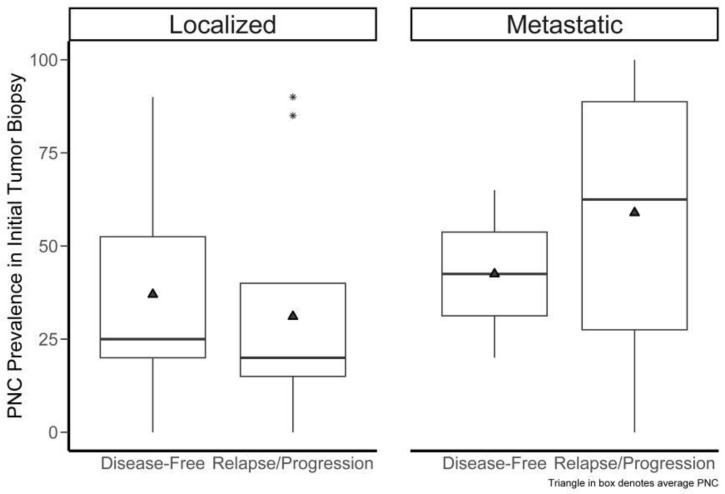
PNC prevalence change in initial tumor biopsy. Patients with metastatic disease and relapse and/or progression had an increased PNC prevalence compared with those who did not have such an event. Triangle is the average PNC per group. Horizontal bar in the box is the median value. The asterisks represent actual data points that are outliers.

An ROC analysis was performed using PNC prevalence and our primary disease-free survival variable, with an AUC of 0.55 (confidence limits: 0.36, 0.72). Youden’s J statistic was 77, which served as the optimal cut-point for attributing “low” (<77) (Figure 1 A,B) and “high” (≥77) PNC (Figure 1C,D). Accordingly, the staining was categorized as diffuse (≥77%) corresponding to high PNC, and not diffuse (<77%), corresponding to low PNC. Hispanics from the US (n = 6, median PNC = 67.5%) had a higher PNC prevalence compared to Caucasians (n = 19, PNC median = 25%) (*p* = 0.017). Patients with metastatic disease at diagnosis had a median PNC prevalence of 62.5% compared to 20% among those with non-metastatic disease at diagnosis (*p* = 0.022) (Table 2). In four patients that had a relapse, immunohistochemistry in the metastatic tissue revealed a PNC prevalence of 90% compared to 37.5% in the initial primary tumor (*p* = 0.011) (Figure 1E). We performed survival analysis using Kaplan–Meier curves and Cox models, finding that a PNC prevalence of ≥77% (high PNC) in primary tumors was predictive of shorter disease-free survival. Stratified Kaplan–Meier survival analysis revealed that most patients with metastatic disease at diagnosis and high PNC had a relapse (Figure 2A), compared to those with non-metastatic diagnosis (Figure 2B). Of the 16 patients with metastatic disease at presentation, those who had relapse/progression had an increase in its prevalence of 23% compared with the group that did not have relapse or progression (Figure 3) (*p* = 0.1024), highlighting the role of the PNC in predicting the outcome of the patients, even in those who present with metastasis at diagnosis (Table 3).

We have compared known prognostic markers such as tumor volume, diameter, etc., between the Mexican/Hispanic patient cohort and the Caucasian cohort, and there were no differences. Only a PNC prevalence of ≥77% was significantly higher in Hispanics (Table 2). Regardless of ethnicity, high PNC prevalence in the local primary tumor was predictive of shorter relapse-free survival using the Cox regression model (Model No. 2 HR 3.26 CL 1.39, 11.51) (Table 3).

### 3.3. Positive and Negative Correlation of miRNAs with High PNC

**Figure 4:**
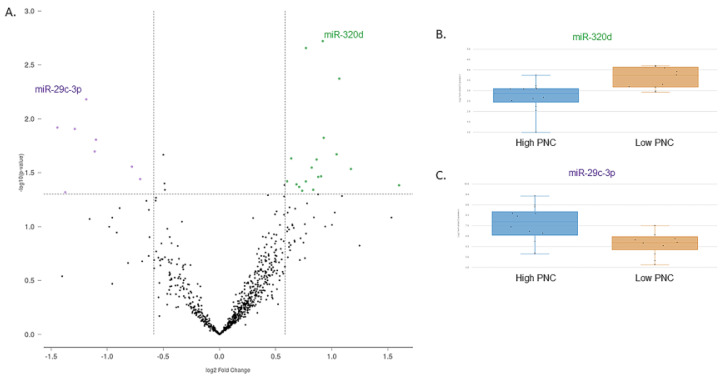
miRNA differential expression data in low versus high PNC: (**A**) volcano plot comparison of EWS tumors (n = 23) with high (n = 12) or low (n = 11) PNC was generated (**A**) and miRNA transcripts with a preset threshold of log2FC of 1.5 is seen on the *x*-axis of the volcano plot, and the negative log of *p*-value are shown on the *y*-axis, with a preset threshold of *p* < 0.05. A total of 26 miRNA transcripts were identified within these parameters, 18 miRNA transcripts were decreased (colored in green) in tumors with high PNC prevalence, and 8 were increased (colored in purple). Two transcripts with the most significant *p*-values include that of miR-320d (**B**) and miR-29c-3p (**C**), which also showed the most significant fold change in high PNC tumors.

Striking miRNA differences were noted in tumors with high versus low PNC, including significant dysregulation of 26 miRNAs (Table 4). Eight miRNAs were significantly upregulated while 18 were downregulated (>1.5-fold change; *p*-value < 0.05) in tumors with high PNC (Table 4). Of these, two transcripts had the most significant change. miR-320d has a decreased fold change of 1.89 in tumors with high PNC with a *p*-value of 0.002, while miR-29c-3p has a 2.28 increased fold change with a *p*-value of 0.006 (Figure 4).

## 4. Discussion

An increased PNC prevalence has been identified in many adult tumors and is correlated with cancer progression, metastasis, and poor overall survival [8,13,23], thus highlighting its prognostic value. This has been demonstrated in various tumor types (prostate, ovarian, breast, pancreatic) in the xenograft mouse models as well as in patient samples. In breast cancer, high PNC prevalence is correlated with large tumor size, lymph node invasion, and histologic high-grade morphology, as well as with poor progression-free survival and worse overall survival [24,25]. The same trend is observed in colorectal tissue samples; PNC prevalence increases with disease stage and progression [11,24]. These facts highlight the role of the PNC in cancer progression, which is related to the presence of oncogenic proteins within the PNC, such as PTB, and their interacting RNAs.

In a similar fashion, our study documented the high PNC prevalence in pediatric EWS. Diffuse staining for PNC or staining ≥ 77% (high PNC) is associated with disease recurrence with pulmonary metastasis and shorter disease-free survival. This is the first study that documents PNC prevalence in a pediatric tumor and highlights the role of the PNC as a prognostic marker. This immunohistochemical study reflects the value of PNC staining in identifying primary tumors that have the potential for metastasis and thus may require more intensive or metastasis-targeted therapy (e.g., with metarrestin). Metarrestin has been shown to disassemble the PNC without affecting normal cell viability [18,26]. In several studies, metarrestin has attenuated cancer growth and suppresses metastasis [12,19,26]. A high prevalence of PNC, as in our study, correlates with an appropriate response to metarrestin. Although not previously tested in EWS, metarrestin is expected to have significant therapeutic potential.

The high prevalence of PNC in Hispanic patients might reflect a biologic basis for the poor prognosis in Hispanics and the racial disparity that has been demonstrated in other studies [23,27]. Of all the tumor parameters listed in Table 2, PNC shows the most significant difference in Hispanics versus Caucasians. Most of the tumors from Hispanic patients (8/12 or 40%) have high PNC in contrast to Caucasians, in whom only 0/19 or 0% of tumors have high PNC.

miRNAs serve diverse functions in the cell and can act as tumor suppressors, while others can act as oncogenes, thus playing an important role in the diagnosis, prognosis, and therapeutics of cancer. Few studies have documented the dysregulation of several miRNAs in EWS. In one study, miR-106b, miR-93, miR-181b, miR-101, and miR-30b were significantly overexpressed, while miR-145, miR-193a-3p, miR-100, miR-22, miR-21, and miR-574-3p were significantly downregulated [28]. These miRNAs regulate many processes related to EWS carcinogenesis, such as the IGF1 pathway and EWS-FLI1 fusion transcript. Our study has disclosed the dysregulation of several miRNAs in association with high PNC, suggesting that miRNA profiling may also serve as another indicator for high PNC. Of these, miR-29c-3p and miR-320d show the most significant association with tumors with a high PNC content. Although not previously studied in EWS, miR-29c-3p is upregulated by p53, leading to the downregulation of miR-29c-3p’s target genes, which are associated with cancer metastasis [29]. Similarly, miR-320d has not been previously studied in EWS but is found to inhibit the tumor growth of colorectal and breast cancers [29,30], reflecting its tumor-suppressive properties. Dysregulation of miRNAs in this study opens a novel avenue for therapeutic intervention. Several miRNA-targeted therapeutics including miRNA mimics and antimiRs have reached clinical development, highlighting the importance of profiling miRNAs in various cancers [31].

## 5. Conclusions

In this study, we demonstrate that the presence of the PNC in pediatric EWS is a clinically relevant predictive biomarker associated with tumor metastasis and shorter disease-free survival. We acknowledge that our sample size is small. However, pediatric Ewing sarcoma is a rare disease, with approximately 350 cases diagnosed annually in the US. The high prevalence of PNC makes it an ideal potential target for novel agents in the future. The PNC can also be used as a predictive biomarker to stratify children at diagnosis, allowing us to identify those at high risk for recurrent disease and potentially provide maintenance therapy after the completion of standard-of-care therapy. PNC can be detected by immunohistochemistry for PTB and by microRNA profiling. PNC analysis by immunohistochemistry is a low-cost tool that may benefit patients in low- and middle-income countries where costly molecular analysis may not be available. The high PNC in Hispanic patients may support biologic differences in tumors, driven by ancestral genetic differences. Hispanics have been grossly underrepresented in biology studies and clinical trials. International collaboration provides an avenue to close the gap in health care disparities globally. 

## Figures and Tables

**Table 1:** Perinucleolar compartment and the distribution of variables in the study population.

		Frequencies n = 40 (%)	PNC ^a^ Prevalence Median [IQR]	*p*-Value ^b^
Sex	Female	22 (55)	32.5 [20, 58.75]	0.763
	Male	18 (45)	37.5 [20, 81.25]	
Age	<10 years	13 (32)	30 [20, 65]	0.999
	≥10 years	26 (65)	35 [20, 77.5]	
Race/Ethnicity	Caucasians	19 (48)	25 [20, 40]	0.212
	Hispanics from USA	6 (15)	67.5 [46.25, 81.25]	
	Mexicans	14 (35)	30 [11.25, 90]	
	Asians	1 (2.5)	85 [85, 85]	
Tumor Volume		n = 31		0.459
	<100 mL	15 (48)	30 [20, 50]	
	≥100 mL	16 (52)	40 [20, 70]	
Tumor Size		n = 31		
	<8 cm	13 (42)	20 [20, 45]	0.271
	≥8 cm	18 (58)	40 [22.5, 77.5]	
Tumor Necrosis		n = 38		
	<90%	24 (63)	30 [20, 65]	0.643
	≥90%	14 (37)	40 [20, 67.5]	
Metastasis at diagnosis	No	24 (60)	20 [18.75, 41.25]	**0.022**
	Yes	16 (40)	62.5 [25, 86.25]	
Progression		n = 38		
	No	28 (74)	22.5 [20, 56.25]	0.079
	Yes	10 (26)	62.5 [28.75, 88.75]	
Relapse		n = 38		
	No	18 (47)	35 [20, 62.5]	0.918
	Yes	20 (53)	35 [18.75, 70]	
Progression and or Relapse		n = 40		
	No	12 (30)	25 [20, 57.5]	0.656
	Yes	28 (70)	37.5 [20, 85]	
Death		n = 40		
	No	18 (45)	35 [20, 58.75]	0.632
	Yes	22 (55)	35 [20, 81.25]	

^a^ Perinucleolar Compartment; ^b^ Wilcoxon Rank-sum test and Kruskal-Wallis. Bold font indicates significant *p*-value.

**Table 2:** Ewing Sarcoma described risk factors by race.

		Hispanics	Caucasians	*p* Value ^a^
Diameter	<8 cm	6	7	0.710
	≥8 cm	6	12	
Volume	<100 cm^3^	7	8	0.473
	≥100 cm^3^	5	11	
Metastasis at diagnosis	No	12	12	0.999
	Yes	8	7	
Primary tumor site	No favorable	11	11	0.752
	Favorable	9	7	
Tumor necrosis	<90%	13	11	0.748
	≥90%	7	8	
High PNC	No	12	19	**0.003**
	Yes	8	0	

^a^ bilateral exact significance by Fisher exact test. Bold font indicates significant *p*-value.

**Table 3:** Multivariate model, Cox regression for disease-free survival.

	Model No. 1		Model No. 2	
	Hazard Ratio	Confidence Limits	Hazard Ratio	Confidence Limits
Metastasis	3.02	1.43, 10.77	3.26	1.39, 11.51
High PNC			2.52	1.10, 7.74

**Table 4:** miRNA significantly dysregulated in high versus low PNC.

Upregulated miRNA	Downregulated miRNA
HSA-miR-490-3p	HSA-miR-1246
HSA-miR-29C-3p	HSA-miR-25-3p
HSA-miR-100-5p	HSA-miR-4488
HSA-miR-30A-5p	HSA-miR-320e
HSA-miR-30A-3p	HSA-miR-483-5p
HSA-miR-152-5p	HSA-miR-320d
	HSA-miR-3195
	HSA-miR-548ar-5p
	HSA-miR-1915-3p
	HSA-miR-601
	HSA-miR-1322
	HSA-miR-500a-5p + HSA-miR-501-5p
	HSA-miR-892b
	HSA-miR-1299
	HSA-miR-1206
	HSA-miR-197-3p
	HSA-miR-198
	HSA-miR-660-5p

## Data Availability

The data presented in this study are available on request from the corresponding author. The data are not publicly available due to patient privacy restrictions.
